# Advances and Challenges of Endoscopic Spine Surgery

**DOI:** 10.3390/jcm13051439

**Published:** 2024-03-01

**Authors:** Daniel Burkett, Nathaniel Brooks

**Affiliations:** Neurosurgery Department, University of Wisconsin Hospitals and Clinics, Madison, WI 53792, USA; burkett@neurosurgery.wisc.edu

**Keywords:** endoscopic spine surgery, spine, endoscopy, navigation, endoscope

## Abstract

The purpose of this paper is to review the data supporting current endoscopic surgical techniques for the spine and the potential challenges and future of the field. The origins of endoscopic spine surgery can be traced back many decades, with many important innovations throughout its development. It can be applied to all levels of the spine, with many robust trials supporting its clinical outcomes. Continued clinical research is needed to explore its expanding indications. Although the limitations of starting an endoscopic program can be justified by its cost effectiveness and positive societal impact, challenges facing its widespread adoption are still present. As more residency and fellowship programs include endoscopy as part of their spine training, it will become more prevalent in hospitals in the United States. Technological advancements in spine surgery will further propel and enhance endoscopic techniques as they become an integral part of a spine surgeon’s repertoire.

## 1. Brief History of Endoscopic Spine Surgery

Approaches for the surgical management of spinal pathology, including disc herniations and spinal stenosis, have evolved significantly since the first lumbar discectomy was described in 1908 [1]. The effort to develop minimally invasive tools and techniques to minimize tissue damage and improve visualization has evolved throughout the history of modern surgery [2]. The evolution of open un-magnified surgery to microscopic surgery has progressed over the last century. Endoscopic approaches in spine surgery represent an advancement of these techniques. Endoscopes such as laparoscopes and cystoscopes have been used consistently in general surgery, urology, and otolaryngology. The utilization of endoscopes for spine surgery has been a more recent development in the latter half of the last century.

The origins of endoscopic approaches to the spine can be traced back to Hijikata and Kambin in the 1970s. Parviz Kambin demonstrated a percutaneous approach for a lateral discectomy using a cannula in 1973. In 1974, Hijikata developed and used tubes to obtain posterolateral access to lumbar disc spaces. He coined this procedure a “percutaneous nucleotomy” [3]. These approaches at the time did not allow for the direct visualization of the spinal canal. The idea of endoscopic direct visualization was applied using a modified arthroscope by Forst and Hausman in the 1980s and later by Kambin using an endoscope. Kambin described a safe anatomical triangular working zone for endoscopic approaches in 1990 [4].

A fully functional endoscopic system (visualization, tissue manipulation, and resection) was used by Anthony Yeung in the 1990s for endoscopic transforaminal approaches [5]. This system used a multichannel, wide-angled endoscope with continuous saline irrigation and underwater bipolar dissection. Multiple endoscopic systems were being developed and utilized in several countries at that time. A description of the successful treatment of far lateral herniated discs using an endoscope through a tubular retractor was published in 1999 by Kevin Foley et al. [6]. A transforaminal approach using bone reamers to open the foraminal window was described in 2005 by Michael Schubert and Thomas Hoogland [7]. In the early 2000s, Sebastian Ruetten applied the technology to interlaminar endoscopic approaches [1].

The continued development of endoscopic techniques has included the use of two separate channels, one working channel and one for the endoscope. This technique is generally termed endoscope-assisted surgery or the unilateral biportal endoscopic (UBE) technique. In 1996, De Antoni described a UBE technique [8]. In addition to an interlaminar approach, a biportal extraforaminal endoscopic approach has also been described [9]. The UBE technique has been supported by good surgical results for the treatment of lumbar stenosis and disc herniation [10].

Today, endoscopic spinal surgery continues to make advances in equipment, techniques, and in the applications of its use. The development of endoscopic spine surgery has advanced significantly for the treatment of many spinal pathologies, but challenges to its widespread adoption remain.

## 2. Endoscope and Instrument Technology

Endoscope systems typically consist of a 20–30 degree rod-lens camera system with a light source, a working channel, and an irrigation channel. Higher-degree scopes are sometimes used for acute working angles such as in the thoracic spine. The interlaminar approach for lumbar discectomy was first performed with a micro endoscopic system in the late 1990s. That endoscope had an outer diameter of 6 mm and a working channel of 2.7 mm. The next generation of endoscopes to follow had an outer diameter of 7.9 mm and a 4.2 mm working channel size. The working channel was large enough to allow the use of endoscopic burrs and punches. The introduction of these instruments allowed surgeons to perform bone resection. The ability to perform bone work under endoscopic visualization expanded the number of pathologies that could be treated [11]. In 2007, the use of these instruments was shown to be effective for the removal of migrated discs [12]. Ruetten later used a 4 mm burr to perform lumbar lateral recess decompression [13].

The third generation of endoscopes had an outer diameter of 9.5 mm and a working channel diameter of 5.6 mm. The increase in the working channel size allowed expanded bony resection and more efficient laminotomies.

The current generation of endoscopes has various endoscopic burrs at their disposal. These burrs have gradually increased in diameter to a working size of 4.5–5.5 mm and include articulating burrs that allow angled drilling. The flexible burrs in conjunction with angled endoscopes allow drilling outside the trajectory of the endoscope working channel. These flexible burrs have demonstrated efficiency when performing endoscopic central canal decompression [14]. Articulating burrs also provide the ability to drill calcified discs in difficult anatomical locations such as the thoracic spine [15]. Curved or angled Kerrison bony punches allow additional wide bony removal. The advancement of endoscopic tools has continued to improve the efficiency of these approaches.

## 3. Current Techniques

### 3.1. Transforaminal Approach

**Figure 1:**
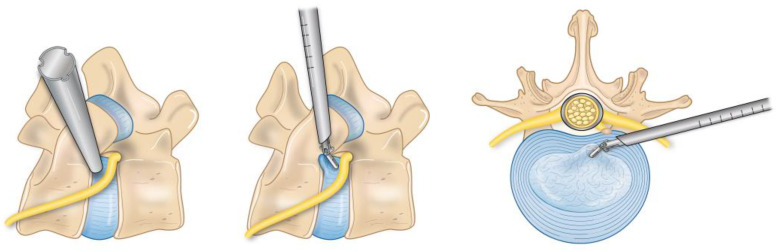
This is a depiction of the foraminal anatomy involved in an endoscopic transforaminal approach.

The endoscopic transforaminal approach is the most traditional uniportal method used in endoscopic spine surgery (ESS) (Figure 1). This approach can be successfully used for the initial treatment of foraminal disc herniations and foraminal stenosis [16]. It is also an option for revision surgeries when trying to avoid a repeat posterior approach. Studies have shown that transforaminal endoscopic surgery and open microscopic surgery did not have significant differences in reoperation and complication rates, but there was less postoperative back pain and a shorter hospital stay in the endoscopic surgery group [17]. An RCT of 143 patients demonstrated that the transforaminal endoscopic discectomy (TED) group had lower affected side leg pain at 2 years compared to the microdiscectomy group. In addition, hospital stays were significantly shorter in the TED group [18]. The anatomical limitations to this approach include the facet joint, pedicle, and the exiting nerve root that can vary with different pathologies.

### 3.2. Lumbar Interlaminar Approach

**Figure 2:**
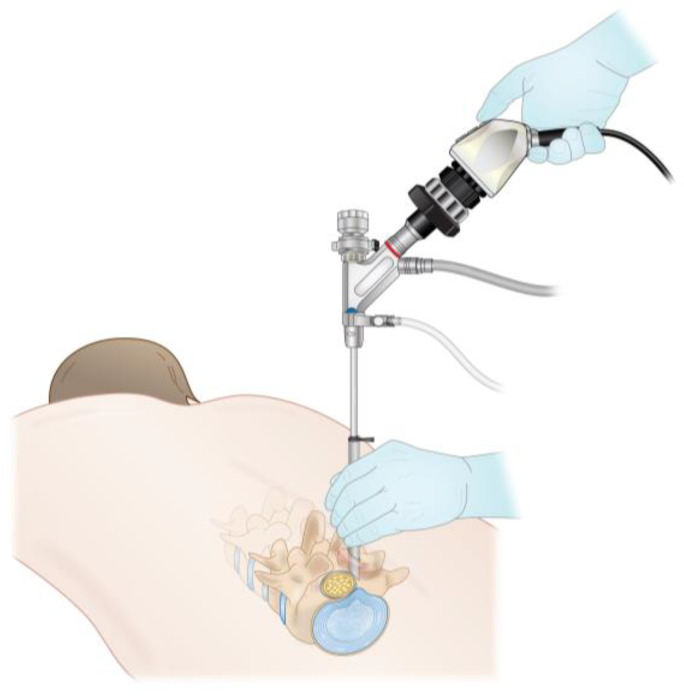
Illustration of the lumbar interlaminar approach.

Sometimes described as the second generation of lumbar endoscopic spine surgery, the interlaminar approach provides more flexibility to an endoscopic spine surgeon’s repertoire (Figure 2). This technique allows the treatment of central and lateral recess canal stenosis and specifically the treatment of L5/S1 disc herniations due to the anatomic constraints of the iliac crest at that level. A meta-analysis reported better results with interlaminar endoscopic lumbar discectomy than transforaminal endoscopic lumbar discectomy in L5/S1 disc herniations [19].

The advantage with the endoscopic interlaminar approach is that the anatomical landmarks are similar to microscopic and traditional anatomic approaches. Additionally, the procedure can more easily be converted to an open operation if needed. Finally, it conserves bony anatomy compared to open surgery, specifically the facet joints. A randomized controlled trial demonstrated similar recovery rates of symptoms between endoscopic and microscopic interlaminar decompression for lateral recess stenosis but found lower rates of revisions and complications with the endoscope [13]. Multiple studies have shown similar decompression, improved clinical outcomes, and shorter hospital stays with endoscopic techniques compared to conventional MIS tubular surgery. These have shown good improvements in Visual Analog Scale (VAS) and Oswestry Disability Index (ODI) scores postoperatively [17,20].

Endoscopic approaches to the lumbar spine have also been argued to serve as treatments for significant central stenosis due to less paraspinal muscle damage and bone resection but sufficient postoperative central decompression. These results are comparable to open surgery for the treatment of multiple levels of lumbar stenosis [21,22].

### 3.3. Cervical Spine Approach

**Figure 3:**
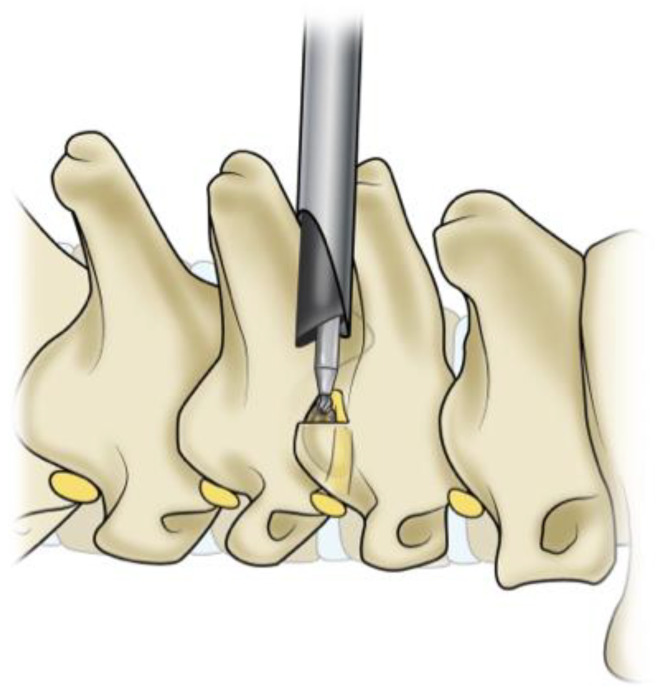
This is an illustration of the posterior cervical foraminotomy approach.

Endoscopic techniques can be used to address both anterior and posterior cervical pathologies and minimize spinal cord manipulation and muscle dissection. Endoscopic posterior cervical foraminotomy is a successful technique for lateral and foraminal disc herniations and has a high success rate of radiculopathy resolution without serious surgical complications [23] (Figure 3). Endoscopic visualization also allows a view of the intervertebral disc not normally seen with conventional open or tubular approaches. After the foraminotomy is performed, the endoscope can be manipulated around the nerve root to remove any parts of the disc causing compression. An RCT showed that endoscopic posterior cervical foraminotomies had similar outcomes to conventional anterior cervical discectomy and fusion (ACDF) [24]. That study noted that the full endoscopic technique had an advantage because it does not require the implantation of hardware. Compared to open foraminotomy, endoscopic surgery also has the advantage of less blood loss, shorter operative times, and shorter lengths of hospital stays [25].

Full endoscopic bilateral decompression for cervical stenosis has been described. Carr et al. reported their first 10 cases with the technique and demonstrated improvement in postoperative Nurick grades and modified Japanese Orthopaedic Association scores without any permanent neurologic complications [26]. Biportal endoscopic laminectomy for the treatment of cervical myelopathy has also been demonstrated and is gaining favor [27]. Full endoscopic and endoscopic-assisted posterior cervical lateral mass screw fixation, C1–2 screw fixation, and laminoplasty procedures have been described without sufficient reports of clinical results.

Long-term clinical results comparing full endoscopic ACDF to conventional ACDF are still needed. Full endoscopic anterior cervical discectomy without fusion in select patients with soft disc herniations, unilateral radiculopathy, and central disc herniations has demonstrated good clinical outcomes [28]. This has been shown to have comparable results with conventional ACDF surgery after a 5-year follow up [29].

### 3.4. Thoracic Spine Approach

Conventional thoracic approaches require extensive muscle dissection and bony resection. Endoscopic approaches allow surgeons to address thoracic disc and spinal canal pathologies directly without significantly disrupting surrounding tissue. Endoscopic transforaminal thoracic discectomy for the treatment of soft disc herniations has demonstrated clinical improvements in VAS and ODI scores after a 5-year follow up [30]. Endoscopic interlaminar, extraforaminal, and transthoracic retropleural approaches for the treatment of disc herniations and thoracic stenosis have shown sufficient spinal cord decompression and successful clinical results [31,32]. The extraforaminal group had a significantly lower complication rate. The treatment of calcified disc herniations via endoscopic approaches has limited data, but these procedures have been performed. Unilateral biportal endoscopic techniques for thoracic fusion surgery have also been described [33]. The same surgical risks and challenges that occur with open thoracic approaches can also challenge endoscopic approaches if the approach is not accurate. This includes injury to the vasculature, lungs, ribs, and postoperative neurological deficits [30].

### 3.5. Lumbar Interbody Fusion

Endoscopic lumbar interbody fusion is gaining favor due to the small incision size and quick postoperative recovery. The approach angle is similar to the current minimally invasive transforaminal lumbar interbody fusion (MIS-TLIF) technique. The instruments are docked and ipsilateral facetectomy and laminotomy are performed. This is then followed by discectomy, endplate preparation, and interbody cage placement [34]. Current indications for endoscopic TLIF are for patients with unilateral foraminal stenosis and mild central stenosis [35]. Other endoscopic TLIF techniques avoid removing the ipsilateral facet altogether in patients who will improve with just indirect decompression [36]. Biportal endoscopic TLIF has shown no significant differences in clinical outcomes compared to conventional MIS-TLIF [37,38]. There are also no reported differences in early and midterm fusion rates between biportal and uniportal endoscopic fusion [39].

In patients with bilateral foraminal stenosis, severe central stenosis, or high-grade spondylolisthesis, unilateral endoscopic lumbar interbody fusion may have limited utility [40]. Endoscopic TLIF has shown promising results, but further long-term studies are needed.

### 3.6. Uniportal and Biportal Approaches

Biportal endoscopy involves the use of an endoscope portal and a working portal. The two channels are placed according to surgeon preference centered on the surgical pathology. A retrospective study compared unilateral biportal endoscopic (UBE) discectomy to open microdiscectomy, and they showed similar results in postoperative pain control, leg pain, and functional disability. UBE had less operative blood loss, shorter hospital stays, and better immediate postoperative back pain [41]. A randomized controlled trial (RCT) published in 2020 compared biportal endoscopic lumbar decompression versus microscopic lumbar decompression and demonstrated equivocal postoperative ODI, EQ-5D, and VAS scores at a 12-month follow up [42].

A comparative analysis studied the differences between biportal endoscopy, uniportal endoscopy, and microsurgery for the treatment of lumbar stenosis via bilateral decompression. Microdiscectomy and biportal endoscopy resulted in more significant dural expansion on postoperative MRI compared to uniportal endoscopy. The mean angle of facetectomy was significantly lower in the biportal endoscopy group compared to the other two. Immediate postoperative VAS scores for back pain were significantly lower in both endoscopy groups compared to the microsurgical group. However, at final follow up, there were no significant differences in the VAS scores for back pain or leg pain and no difference in ODI for all three groups [43].

When comparing uniportal and biportal endoscopic surgery, both procedures demonstrated similar efficacy, but the operative time was shorter and central canal decompression was improved in the UBE group [44].

## 4. Advantages of Endoscopic Spine Surgery

Endoscopic approaches are a great option because of their small incision size and minimal muscle dissection while still providing successful clinical outcomes. Several randomized controlled trials have demonstrated the clinical success of these approaches (Table 1). Transforaminal endoscopic discectomy (TED) has been shown to lead to better postoperative back pain, a shorter hospital stay, and a faster overall recovery compared to conventional open microdiscectomy [45]. An RCT published in 2022 compared TED to conventional open microdiscectomy for patients with at least six weeks of radiating leg pain. The study showed that patients randomized to TED had a significantly lower postoperative VAS score for leg pain compared to conventional open microdiscectomy. There was also less blood loss, shorter hospital stays, and less back pain in the TED group [46]. A total of 125 patients in this study were part of the “learning curve” group for surgeons learning how to perform PTED. Surgeons can still have successful outcomes while learning endoscopic techniques.

Lumbar stenosis is also a target of endoscopic treatment. A 2009 RCT showed that full endoscopic interlaminar decompression had similar clinical outcomes but with significantly lower complication rates compared to microsurgical decompression [13]. Compared to tubular minimally invasive techniques, endoscopic ULBD has shown reductions in the hospital length of stay, 1-year postoperative leg pain, and back pain disability [47].

**Table 1:** Table of randomized controlled trials comparing endoscopic spine approaches to open approaches.

Study	Year	Study Location	Approach Technique	Outcomes Measured
Gibson et al. [18]	2017	UK	Lumbar Transforaminal	Affected side leg pain, revision rate, complication rate, functional outcomes, hospital stay
Gadjradj et al. [46]	2022	Netherlands	Lumbar Transforaminal	VAS-leg score, blood loss, hospital LOS, complication rate, ODI, VAS back pain
Ruetten et al. [48]	2008	Germany	Lumbar Transforaminal and Interlaminar	Back pain, leg pain, work disability, complication rates, recurrence rate
Ruetten et al. [13]	2009	Germany	Lumbar Interlaminar Decompression	Leg pain, complication rate, revision rate
Ruetten et al. [49]	2009	Germany	Lumbar Transforaminal and Interlaminar	Recurrent herniation
Ruetten et al. [24]	2008	Germany	Posterior Cervical Interlaminar	Arm pain, neck pain, recurrence rate

### 4.1. Revision Surgery

Surgery for the treatment of recurrent spinal pathologies comes with an increased risk of complications including durotomies and infection due to surrounding scar tissue and dural adhesions [50]. A transforaminal endoscopic approach can provide an advantage in this population with targeted approaches through new tissue. A prospective study by Hoogland et al. looked at 262 patients who underwent transforaminal endoscopic lumbar discectomy for recurrent disc herniation. At the 2-year follow up, more than 95% of patients had reported good outcomes and there was a 3.8% complication rate with no infections or durotomies. Of those patients, 4.7% reported a third recurrent disc herniation [51].

### 4.2. Obesity

Endoscopic spine surgery does not require extensive tissue dissection and provides an advantage in obese patients. A study of 41 patients with a body mass index > 30 kg/m^2^ who underwent endoscopic lumbar decompression procedures showed that they were able to achieve significant improvements in pain and disability without high amounts of blood loss or postoperative complications [52]. The mean operative time was shorter in endoscopic discectomy patients compared to those who underwent open microdiscectomy in obese patients with a BMI > 30 kg/m^2^ [53].

### 4.3. Surgical Site Infection Reduction

Full endoscopic spine surgery has been shown to have a significantly lower risk of surgical site infections (SSIs). A retrospective multicenter cohort study compared 1277 noninstrumented full endoscopic spine surgery (FESS) cases compared to 55,882 nonendoscopic NSQIP cohort patients. In the matched data, the SSI rates for nonendoscopic and endoscopic patients were 1.2% and 0.001%, respectively, which was a 16-times reduction [54]. The rate of infection with traditional microdiscectomies is low, but as full endoscopic surgical techniques are applied broadly, the reduction in SSIs can have a larger effect.

### 4.4. Anesthesia Techniques

Endoscopic spine surgery offers flexibility with anesthesia techniques. Due to the small incision size, shorter operative time, and minimal blood loss during surgery, endoscopic spinal surgery is an ideal candidate for local anesthesia and monitored anesthesia care (MAC). A meta-analysis looked at the differences between local anesthesia compared to general anesthesia in percutaneous interlaminar endoscopic discectomy. The local anesthesia group had shorter hospital length of stays and less hospital costs but similar postoperative pain scores, complications, and operative times [55].

MAC with local anesthetic blocks avoids the risks of general anesthesia and maximizes the potential recovery of the patient. MAC leads to more rapid discharges, fewer anesthesia complication risks, and shorter average postoperative hospital stays [56]. Surgeons have been successfully performing awake endoscopic transforaminal lumbar interbody fusions on select surgical candidates [36]. Pathologies such as high-grade spondylolisthesis or multiple-level surgeries that require longer operative times will likely require general anesthesia [36]. Awake surgeries give patients a stronger chance of discharging faster after surgery, reduce the chances of hospital-related complications, and save costs for hospitals.

### 4.5. Ambulatory Center Compatibility

More hospital systems and surgeons have focused on the development of ambulatory surgery centers (ASCs) due to the overall lower costs involved and the ability to focus on outpatient and short-hospital-stay procedures. Endoscopic procedures, given their small incision size and ability to be performed without general anesthesia, are ideal for these settings and have been shown to be successful in ASCs. Lewandrowski evaluated 1839 patients undergoing transforaminal endoscopic decompression surgery for lumbar foraminal and lateral recess stenosis in an ASC over 10 years. In total, 82% of those patients had good or better outcomes without any major approach-related complications [16]. Selecting patients that are ideal for these outpatient surgical settings is integral to its success.

### 4.6. Future Indications

Endoscopic spine approaches are well suited to treat disc herniations and stenosis from ligamentum hypertrophy, osteophytes, and other compressive pathologies at all levels of the spine. Pathologies such as spinal infections and neoplasms prove to be a stronger challenge. A few case reports have demonstrated the feasibility of endoscopic techniques to treat osseous lesions of the spine and extradural tumors [57,58]. The endoscopic resection of tumors is limited to benign lesions due to significant tissue loss with continuous irrigation in endoscopy and limited exposure. At this time, there are insufficient levels of evidence to definitively recommend the endoscopic resection of spinal neoplasms, and long-term prospective data are needed.

The endoscopic treatment of spinal infections has been reported in small retrospective reviews and case studies. The endoscopic debridement of spondylodiscitis has demonstrated 54.2–90% success in microorganism identification but up to a 33% rate of treatment failure [59]. A retrospective review of 20 patients undergoing endoscopic debridement and drainage of surgical infections after lumbar-instrumented fusion demonstrated an 85% rate of successful bacterial identification and infection control in 65% of patients [60]. Spinal infections pose surgical variability depending on the amount of scar present and the consistency of the infection, and the ability to convert to open debridement is necessary. Future prospective studies may demonstrate the benefit of the endoscopic treatment of spinal infections.

## 5. Limitations in Endoscopic Spine Surgery

### 5.1. Anatomical

Spinal endoscopy has several technical and economic challenges facing its widespread adoption. Due to its very selective and small area of focus with the endoscope, a pathology requiring the treatment of greater than three levels or deformity correction is not currently recommended.

Foraminal anatomy can also present a challenge during surgery. The transforaminal approach works within Kambin’s triangle, where the exiting nerve root is usually in the cranial aspect of the foramen just under the superior vertebra’s pedicle, traveling ventrally and caudally. The foraminal height and diameter decrease in a rostral to caudal direction down the lumbar spine. This changes the location of the inferior border of the nerve in relation to the disc space. It is cranial to the superior aspect of the intervertebral disc at L2/3 and L3/4 and inferior to it at L4/5 and L5/S1. Anatomical variations can be a significant barrier to becoming comfortable with the surgical anatomy. The significant manipulation or retraction of the exiting nerve root can lead to postoperative dysesthesia and radiculitis [61,62].

### 5.2. Complications

A 2019 retrospective study of 1839 patients showed that overall complications after transforaminal endoscopic discectomy was one magnitude lower than the microdiscectomy complication rate [16]. A meta-analysis of six RCTs showed that endoscopic lumbar discectomy had a 50% reduction in overall complications compared to traditional lumbar microdiscectomy [63]. Complications such as durotomies, neural injury, and damage to the facet joints all occur in endoscopic spine surgery and will happen more frequently when first starting to learn and practice the techniques. As a surgeon becomes more experienced, the complication rate decreases and can be comparable to similar MISS procedures [16]. Retrospective survey data of 64,470 lumbar endoscopy cases resulted in 689 dural tears with a durotomy incidence of 1.07% [64]. Small durotomies can be repaired with a dural graft and dural glue or left unrepaired due to surrounding tissue pressure [65]. Nonpenetrating clips have demonstrated the feasibility of dural closure during biportal endoscopic procedures but are not used during uniportal procedures due to the device size [66]. Classic suture closure through an endoscope is difficult, but the advancement of dural closure technology is helping make dural repair during endoscopic surgery more feasible [67].

### 5.3. Capital Expenditure and Reimbursement Challenges

In order to establish a successful endoscopic program, its economic value needs to be proven to hospital systems. This includes keeping capital expenditures low, proving the cost effectiveness of the cases performed, and making sure insurance companies reimburse endoscopic spine cases. The initial capital expenditure of endoscopic equipment in North America has been reported to be as high as USD 350,000 [68]. In addition, a study found that the equipment costs per case can average about JPY 158,000, equivalent to about USD 1080 [69].

Spinal endoscopy is associated with advantageous healthcare and societal costs [70]. Endoscopic discectomy, on average, enables about USD 8000 in cost savings per 1 QALY compared to microdiscectomy [71]. A study looking at the cost-effectiveness of PTED compared to open microdiscectomy for lumbar disc herniation demonstrated that significant postoperative improvements in leg pain and quality-adjusted life years (QALYs) were found in the PTED group at 12 months. Surgical costs were higher for PTED compared to open microdiscectomy, but total societal costs were lower for PTED. PTED was found to be more cost effective overall compared to open microdiscectomy from the societal perspective [72]. This study found that surgery costs were about EUR 4500 per patient for PTED. It can take years before the initial investment into endoscopic spine surgery can pay off with its benefits to the hospital and society. These benefits include faster discharges and a reduction in overall complications and their costs [73].

Insurance authorization is another hurdle for spinal endoscopy adoption. Reimbursement is difficult due to strict requirements for proving the cost–benefit of endoscopic surgery. A retrospective analysis of 1839 cases of patients with lumbar stenosis demonstrated that in 66% of cases, the radiologist’s preoperative MRI report and the intraoperative endoscopic visualization of lumbar compression did not correlate. They also found that the accuracy of the reports increased when the operating surgeon graded the same imaging findings preoperatively [74]. These differences in radiographic reports can affect patient selection and increase the difficulty of insurance authorization for endoscopic surgery.

The first Current Procedural Terminology code (number 62380) for endoscopic spine surgery was first released in 2017. This code covers only lumbar endoscopic decompression of the spinal cord but does not differentiate between different types of decompression or if a discectomy is performed. As endoscopic spine surgery gains more favor, coding will need to cover the various types of procedures it can be applied to.

### 5.4. Training Surgeons and the Endoscopic Learning Curve

Interest in endoscopic spine surgery has been increasing over the last 10 years, as demonstrated by the increase in publications, but specific training in this area is not common in residency or spine fellowship curricula [75]. Only a few large academic institutions across the country advertise endoscopic spine surgery training, but this number continues to grow every year. At institutions without endoscopic spine exposure or places looking to increase their exposure, it may be beneficial to set up training models and simulators. Virtual reality simulators can provide detailed visual feedback but are expensive and may lack tactile feedback. Low-cost training models have been described as effective alternatives for teaching endoscopic techniques [76].

Formal training besides residency or fellowship is very limited. Even if a surgeon is experienced in endoscopic surgical work via joint arthroscopy, endonasal skull base work, or intraventricular endoscopy, the anatomy and instrument handling is different with spinal endoscopy. Current practicing surgeons and residents without case exposure are learning the techniques during cadaver courses where they have only a day or two to practice the techniques [77]. Studies suggest that it takes about 70 cases for training surgeons to produce good results [78]. A study looked at the learning curve for two experienced surgeons in practice for a combined 20 years before entering apprenticeships with master endoscopic surgeons. They reported the learning curve for endoscopic decompression was 15 cases. These same surgeons had slightly worse outcomes using the endoscopic technique compared to their traditional decompression technique [79]. The difference in results became narrower with time.

Virtual reality (VR) and mixed-reality simulators could provide a great teaching tool for educating new adopters of endoscopic surgery. VR preoperative planning for endoscopic approaches has been shown to improve accuracy and shorten operative times [80].

## 6. Endoscopic Enabling Technology

### 6.1. Navigation Techniques

Assessing the intraoperative target spinal pathology through endoscopic visualization is difficult. Intraoperative fluoroscopy is currently used to pinpoint the trajectory and target but only provides two-dimensional images. In addition, there is radiation exposure to the operating room staff and patient.

Intraoperative imaging can create three-dimensional images that are displayed on an intraoperative monitor. With current technology, operative tracking tools can be synced with the interactive 3D reconstruction and anatomy can be confirmed in real time. This technology has allowed for more accurate traditional pedicle screw placement and a decrease in radiation exposure to the operating room staff [81,82]. Intraoperative navigation can be effectively used to assist endoscopic surgery with instrument accuracy and the placement of hardware and devices [83].

Most systems use optical tracking requiring a line of sight between the camera and instruments. Electromagnetic (EM) navigation does not require line-of-sight tracking and has been shown to reduce endoscope docking and the total operative time compared to conventional fluoroscopy in a randomized controlled trial. In that same study, patients had similar functional outcomes between the groups [84]. Improvements in navigation techniques will continue to help endoscopic spine surgery expand its applications and assist new learners in adopting the techniques.

### 6.2. Augmented Reality

Augmented Reality (AR) has begun to gain traction in spine surgery because of its ability to show surgeons computer-generated anatomy overlaying a patient’s intraoperative anatomy. The ability to show surgeons their anatomical trajectories through their own point of view may assist with the adoption of endoscopic approaches and intraoperative localization. The current generation of equipment is not used in many operating rooms, but as it becomes more mainstream, it may find a role in endoscopic spine surgery.

### 6.3. Robotic Assistance

Intraoperative robotic assistance is gaining traction in spine surgery with the goal of creating safe and consistent outcomes. Navigated robotic assistance provides a stable cannula for the surgeon to place their tools through for pedicle screw placement. It has been successful when used for pedicle screw fixation and has been shown to have improved outcomes compared to freehand and fluoroscopic-assisted techniques [85,86].

In endoscopic approaches, navigated robot assistance could theoretically be used to accurately position endoscopes and then hold the scopes in a stable position throughout surgery. It has been used as an assistive tool during endoscopic TLIF surgery to place pedicle screws and help plan the ideal trajectory for endoscopic discectomy [87]. Robotic arms have been used to provide a stable trajectory and a depth stop for the endoscope and the endoscopic drill [88]. Robot-assisted endoscopic surgery is still new and needs further studies to determine its utility.

### 6.4. Visual Technology

With current video technology, the surgeon views the operative endoscopic view on a two-dimensional (2D) screen. The screen resolution varies depending on the quality of the monitor and the endoscopic equipment. Newer endoscopes are adopting 4K resolution technology in conjunction with 4K compatible monitors. The high fidelity provides a crisper image attempting to mimic the use of a microscope. The anatomy viewed within the endoscope can be difficult to translate from a three-dimensional (3D) working space on a 2D screen. Three-dimensional-capable screens could improve depth perception, allowing for the easier distinction of intraoperative pathology and neural structures [89].

## 7. Conclusions

Endoscopic spine surgery has its roots in early non-visualized percutaneous procedures. Advancements in techniques and technology have allowed the field to expand its capabilities and success. The robust data supporting its clinical outcomes and cost effectiveness will fuel its continued implementation in the operating room. There are still many challenges that face this constantly evolving area, but improvements in technology and surgeon education will expand its growth. As the drive to improve the cost and quality of care provided to patients continues, innovations will propel this growing field forward.

## Data Availability

No new data were created or analyzed in this study. Data sharing is not applicable to this article.
